# Development of a High-Speed Precision Ultrasonic-Assisted Spindle for Ultra-Precision Optical Mold Machining

**DOI:** 10.3390/s24227145

**Published:** 2024-11-07

**Authors:** Dingwen Wang, Qiu Hong, Shaohui Yin

**Affiliations:** 1 College of Mechanical and Vehicle Engineering, Hunan University, Changsha 410082, China; wangdingwen@hnu.edu.cn; 2 National Engineering Research Center for High Efficiency Grinding, Hunan University, Changsha 410082, China; 3 School of Intelligent Manufacturing and Electronic Engineering, Wenzhou University of Technology, Wenzhou 325035, China; hongqiu@hnu.edu.cn

**Keywords:** ultrasonic-assisted grinding, optical molds, grinding spindles, air turbine, aerostatic bearing

## Abstract

Ultrasonic vibration-assisted grinding is a critical method for machining ultra-hard optical molds. However, current ultrasonic-assisted grinding spindles, as essential foundational equipment, face limitations in maintaining ultra-high rotational speed, high precision, and a compact structure during ultrasonic operation. This study presents a novel ultra-precision ultrasonic-assisted high-speed aerostatic spindle for grinding ultra-hard optical molds, developed through theoretical calculations, FEM, and CFD simulations. The spindle features a simple and compact design (φ60 mm outer diameter × 194 mm length), operates at an ultrasonic frequency of 41.23 kHz, and is driven by an impulse turbine providing torque up to 50.4 N•mm, achieving speeds exceeding 40,000 r/min. Aerostatic bearings provide axial and radial load capacities of 89 N and 220 N, respectively. The results demonstrate that the proposed high-speed precision ultrasonic spindle exhibits both feasibility and potential for practical application.

## 1. Introduction

With the rapid development of industries such as optoelectronic communications, optics, automotive, medical endoscopy, bioengineering, and aerospace, the demand for aspheric optical lenses with submicron surface precision has significantly increased. Currently, the hot-press molding technology for optical glass components enables the mass production of aspheric lenses with low cost and high stability [1]. However, the molds used for hot-pressing optical glass must meet stringent requirements, including surface form accuracy with a peak-to-valley (P–V) value of 100–300 nm, nanometer-level surface roughness, and minimal subsurface damage. These molds are typically made from hard and brittle materials such as WC or SiC. Due to the high hardness and low fracture toughness of these materials, along with the complexity of the required processing equipment, achieving an ideal high-precision, high-efficiency machining method has long remained a challenge in the industry [2,3].

Currently, various methods are employed for machining hard and brittle materials, including grinding [4], electrochemical machining [5], laser machining [6], electron beam machining [7], ion beam machining [8], ultrasonic machining [9], electrochemical machining [10], and rotary ultrasonic machining [11,12]. Among these, ultrasonic and rotary ultrasonic machining techniques superimpose micron-scale high-frequency elliptical vibrations onto the tool or workpiece, leading to periodic contact and separation between them [13,14,15]. This approach reduces the instantaneous cutting thickness and periodically reverses the friction direction between the tool rake face and chips [16,17], demonstrating significant advantages in reducing cutting forces, minimizing tool wear [18], and improving surface quality [19]. Consequently, ultrasonic-assisted methods are considered effective for machining hard and brittle materials [20].

With the continuous advancement in machine tool development and the maturation of piezoelectric ceramics as transducer technology, companies such as DMG (Saibach, Germany) [21], AREUSE (Saitama, Japan) [22], and TAKESHO (Fukuoka, Japan) [23] have developed various rotary ultrasonic machining systems for a range of applications, integrating these systems as machine tool attachments. However, these rotary ultrasonic systems are primarily integrated into machining centers, limiting their suitability for high-precision micro-grinding of ultra-hard materials. These systems struggle to achieve both the high rotational speeds and effective transmission and coupling of ultrasonic energy required for such demanding applications.

To address the growing demand for ultrasonic-assisted micro-machining, improve machining efficiency, and reduce the complexity of ultrasonic-assisted equipment, Japanese researchers such as Tsunemoto Kuriyagawa developed an ultrasonic spindle operating at a frequency of 25 kHz with a maximum amplitude of 20 μm, achieving a rotational accuracy of 0.7 μm. However, the spindle’s speed was limited to only 1000 r/min [24]. In 2011, Japanese researcher Hidenari Kanai and colleagues developed another spindle with an ultrasonic frequency of 41 kHz and a maximum speed of 40,000 r/min, though its maximum amplitude was limited to 2.5 μm [25]. To further enhance the rotational speed and radial precision, in 2019, Sebastian Greco from Germany proposed a novel air-driven ultrasonic spindle design based on magnetostrictive effects, capable of reaching an ultrasonic frequency of 16 kHz and a maximum speed of 100,000 r/min [26]. However, no mature ultrasonic spindle product has been introduced into the market to date.

To simplify the spindle structure, facilitate commercialization, and achieve higher rotational speeds with minimal radial runout under ultrasonic vibration, we have developed a novel high-speed ultra-precision ultrasonic spindle. This study investigates its load-bearing characteristics, torque, ultrasonic frequency, and amplitude performance.

## 2. Spindle Design Concept

**Figure 1:**
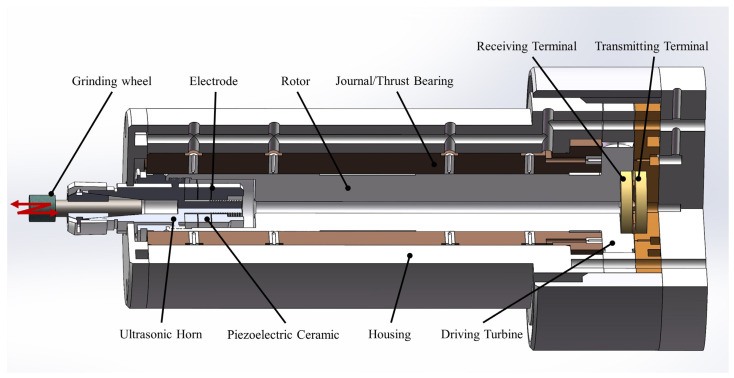
Sectional view of the ultrasonic-assisted high-speed air-bearing spindle.

The spindle is designed to achieve a rotational speed exceeding 40,000 r/min and a resonant frequency of at least 40 kHz. To enhance the rotational speed and precision, the spindle rotor is driven by an air turbine and supported by aerostatic bearings. To simplify the spindle structure and reduce manufacturing complexity and costs, the turbine is integrated with the rotor. Additionally, two radial aerostatic bearings and one thrust aerostatic bearing are designed as a single unit to improve the alignment accuracy. The design and arrangement of the spindle and its components are illustrated in Figure 1.

The ultrasonic vibration of the spindle is generated by piezoelectric ceramics. An ultrasonic generator converts a 220V AC power supply into an ultrasonic-frequency electrical signal, which is transmitted to a sandwich-type piezoelectric transducer through non-contact power transmission. The transducer converts the ultrasonic electrical oscillations into ultrasonic-frequency mechanical vibrations, which are amplified by the horn and tool, producing effective ultrasonic mechanical vibrations at the tooltip. The piezoelectric ceramic material is PZT-8, with an inner diameter of 6 mm, an outer diameter of 15 mm, and a thickness of 4 mm. Due to its low dielectric constant, low electromechanical coupling coefficient, and low piezoelectric constant, PZT-8 exhibits superior tensile strength and stability, as well as a high mechanical quality factor, making it particularly well-suited for applications that require high mechanical amplitude. The mechanical properties of PZT-8 are shown in Table 1 [27].

## 3. Spindle Structure CFD Simulation and Optimization

To reduce the whirl effects generated by the air turbine during high-speed rotation, numerical calculations and finite element simulations were employed to study and optimize the spindle turbine structure. Additionally, CFD simulations were used to analyze the characteristics of the radial and axial gas-lubricated aerostatic bearings in the air-floating spindle.

### 3.1. Mathematical Model

The jet within the nozzle is modeled as a steady adiabatic expansion process. According to Bernoulli’s equation, the average diameter of the turbine is determined by Equation (1) [28]:

(1) $$D_{\mathrm{ave}}=\frac{60x\sqrt{2kRT_{0}^{*}\left(1-{\left(P_{0}^{*}/P_{2}\right)}^{\left(1-k\right)/k}\right)/\left(k-1\right)}}{\pi n_{T}}$$

where $n_{T}$ represents the turbine rotational speed, x denotes the loss coefficient (chosen within the range of 0.2 to 0.4), k is the adiabatic index of the gas, R is the gas constant, $T_{0}^{*}$ refers to the total temperature at the turbine inlet, $P_{0}^{*}$ is the supply pressure, and $P_{2}$ indicates the pressure in the turbine chamber or back pressure.

The height of the turbine blade is calculated as

(2) $$h=0.75\times 10^{-6}\times D_{ave}\times \frac{n_{st}^{2}\left(k+1-\left(k-1\right){\left(\varphi C_{ad}/a_{cr}\right)}^{2}\right)}{\varepsilon {\left(u/C_{ad}\right)}^{2}\varphi \sin\alpha_{1}\left(k+1-\left(k-1\right){\left(C_{ad}/a_{cr}\right)}^{2}\right)}$$

where ε represents the local flow coefficient, and φ is the nozzle velocity coefficient, selected within the range of 0.92 to 0.96. $\alpha_{1}$ denotes the nozzle exit angle, chosen from 15° to 20°. $q_{mT}$ indicates the mass flow rate of the gas, $C_{ad}$ is the adiabatic velocity at the nozzle exit, $a_{cr}$ represents the critical velocity at the nozzle exit, and $n_{st}$ refers to the turbine-specific speed.

The formula for calculating the mass flow rate $q_{mT}$ of an airflow is

(3) $$q_{mT}=N_{T}\left(k-1\right)/\left(2\eta_{T}kRT_{0}^{*}\left(1-{\left(P_{0}^{*}/P_{2}\right)}^{\left(1-k\right)/k}\right)\right)$$

where $\eta_{T}$ denotes the turbine efficiency, which ranges from 0.68 to 0.75.

The formula for determining the turbine specific speed $n_{st}$ is

(4) $$n_{st}=193.3\omega \sqrt{q_{mT}RT_{0}^{*}/\left(P_{0}^{*}{\left(1-\frac{k-1}{k+1}{\left(C_{ad}/a_{cr}\right)}^{2}\right)}^{1/\left(k-1\right)}\right)}/{\left(kRT_{0}^{*}\left(1-{\left(P_{0}^{*}/P_{2}\right)}^{\left(1-k\right)/k}\right)/\left(k-1\right)\right)}^{3/4}$$

The formula for calculating the adiabatic exit velocity $C_{ad}$ of the nozzle is

(5) $$C_{ad}=\sqrt{2kRT_{0}^{*}\left(1-{\left(P_{0}^{*}/P_{2}\right)}^{\left(1-k\right)/k}\right)/\left(k-1\right)}$$

The angle of the gas flow at the turbine inlet is

(6) $$\beta_{1}=\arctan\frac{\sin\alpha_{1}}{\cos\alpha_{1}-u/C_{ad}}$$

where u represents the tangential velocity at the mean diameter of the turbine.

The absolute exit angle of the gas flow at the turbine outlet is

(7) $$\alpha_{2}=\arctan\frac{\psi \omega_{1}\sin\beta_{2}}{\psi \omega_{1}\cos\beta_{2}-u}$$

where $\omega_{1}$ denotes the relative velocity of the gas at the turbine inlet, and ψ represents the velocity coefficient for relative motion, which typically ranges from 0.88 to 0.92 and, for simplification in turbine design, is generally approximated as $\beta_{2}$ = $\beta_{1}$.

The relative velocity of the gas $\omega_{1}$ at the turbine inlet is calculated using the following formula:

(8) $$\omega_{1}=\sqrt{{\left(\varphi C_{ad}\cos\alpha_{1}-u\right)}^{2}+{\left(\varphi C_{ad}\sin\alpha_{1}\right)}^{2}}$$

The minimum cross-sectional diameter of the nozzle is

(9) $$d_{\min}=\sqrt{\frac{4q_{mT}\sqrt{RT_{0}^{*}}}{\pi n_{c}P_{1cr}^{*}\sqrt{k{\left(2/\left(k+1\right)\right)}^{\left(k+1\right)/\left(k-1\right)}}}}$$

### 3.2. Simulation and Optimization of the Turbine Structure

Since the cutting force in ultra-precision grinding is typically less than 5 N [29,30,31], the torque generated when using a grinding wheel with a diameter of 8 mm is less than 20 N•mm. Therefore, to achieve high rotational speeds and compensate for the frictional power losses in the aerostatic bearings, the turbine rotor must generate sufficient torque. The frictional power losses in the spindle shaft and thrust bearings can be calculated using Petroff’s equation [32].

**Figure 2:**
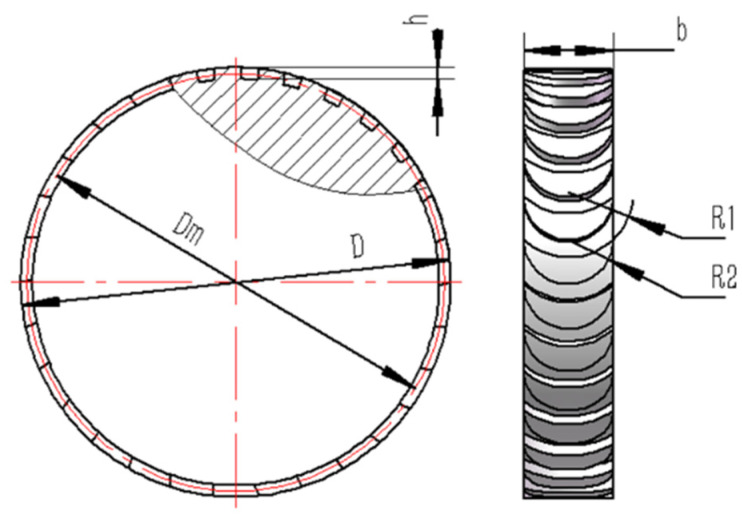
Geometrics of the air turbine.

**Figure 3:**
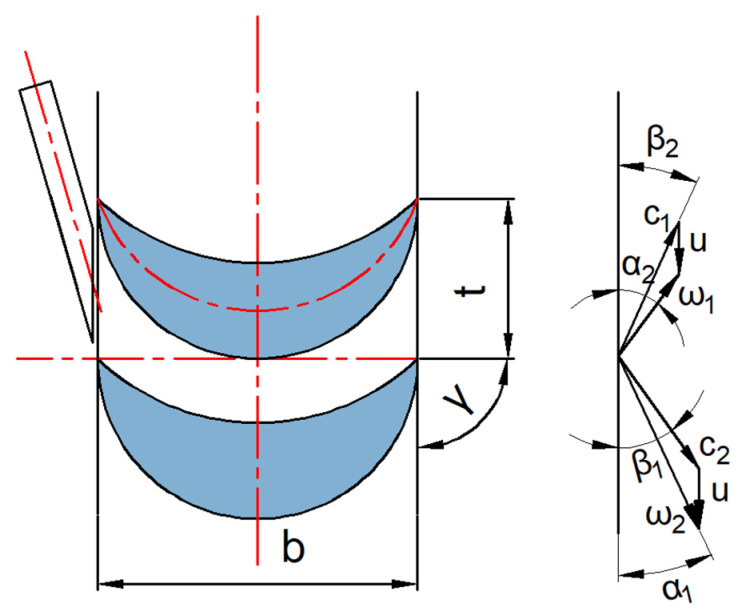
Schematic of the driving turbine and nozzle.

**Figure 4:**
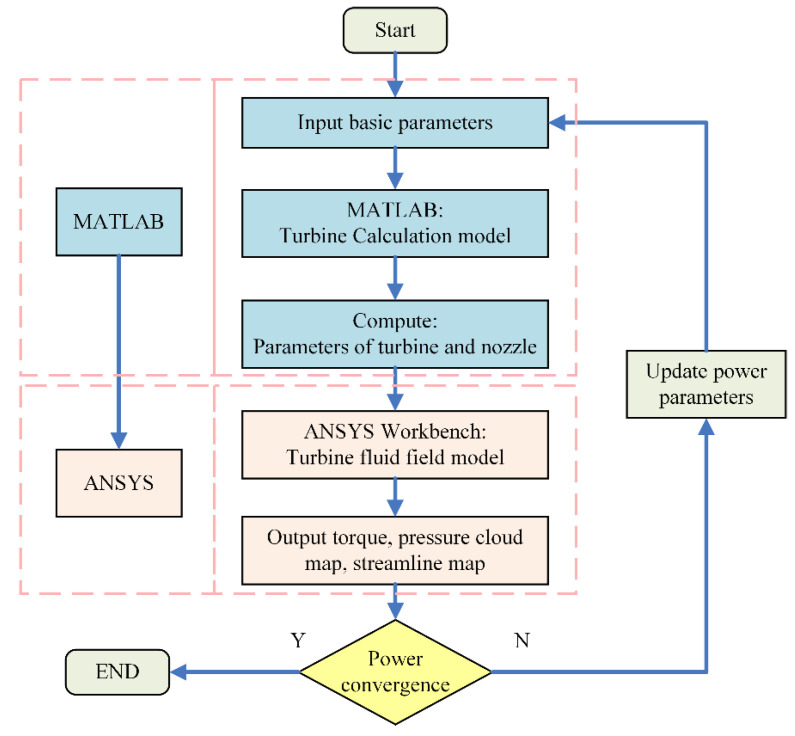
Flowchart for solving the turbine and nozzle parameters.

The basic structure of the turbine is illustrated in Figure 2. It is an axial impulse turbine with a simple design that is easy to manufacture and capable of achieving high rotational speeds with low rotational error. The turbine is driven by high-velocity airflow that is expanded and accelerated through nozzles, which are evenly distributed along the turbine’s circumference. To design and optimize the geometric parameters of the turbine and nozzles, as shown in Figure 3, theoretical analysis and finite element simulations were employed to model the internal airflow of the turbine and calculate the effects of geometric parameters on its aerodynamic characteristics. The simulation process is illustrated in Figure 4. First, MATLAB was used to calculate the geometric parameters of the turbine and nozzles. Next, a geometric model of the turbine flow passage was constructed based on the calculated parameters. Finally, the model was imported into ANSYS Workbench to analyze the internal airflow characteristics of the turbine, and the turbine and nozzle structures were optimized based on the analysis of their aerodynamic properties.

**Figure 5:**
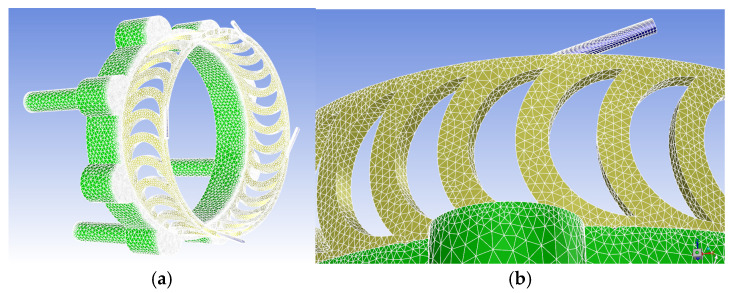
Meshing of the aerodynamic turbine flow passage: (**a**) the overall mesh diagram of the aerodynamic turbine flow channel region; (**b**) a local mesh diagram of the aerodynamic turbine flow channel region.

Given the presence of both rotating and stationary domains within the fluid region, along with the structural complexity, the external intake and exhaust ducts were excluded to simplify the simulation model. The fluid region was divided into three parts: the nozzle, the rotating blade domain, and the exhaust pipe. Each part was meshed separately to improve the mesh quality. After meshing, the nozzle, rotating blade domain, and exhaust pipe were assembled in Fluent to form the complete turbine flow channel model, as shown in Figure 5. The nozzle was meshed with structured grids, while the rotating blade domain and exhaust pipe were meshed with unstructured hexahedral grids, resulting in a total of approximately 5.7 million mesh elements. The Multiple Reference Frame (MRF) model was used to set the rotational speed for the rotating blade domain, with the nozzle inlet defined as the pressure inlet boundary and the exhaust outlet as the pressure outlet boundary at 101,325 Pa. Since the k-ω SST turbulence model is one of the best-performing eddy viscosity models [33], it was selected as the turbulence model. The convergence criteria were set such that the residuals for velocity, turbulent kinetic energy, and the dissipation rate were less than 0.001, and the mass residual was below 0.5%.

**Figure 6:**
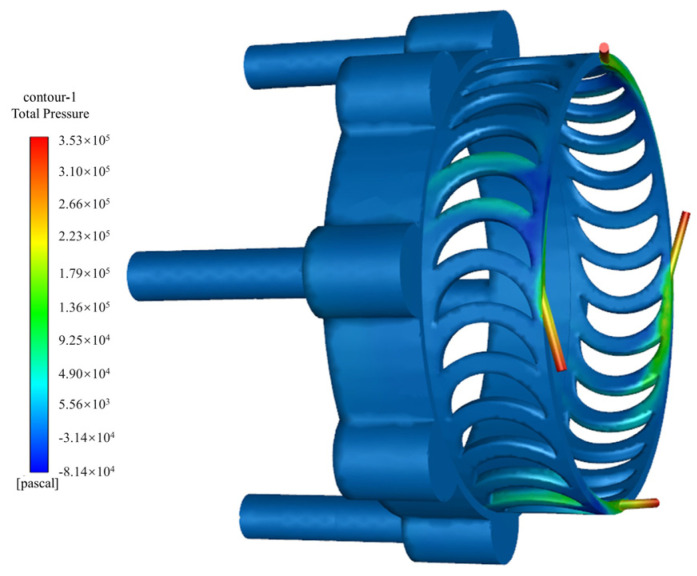
Pressure distribution of airflow within the turbine flow passage.

**Figure 7:**
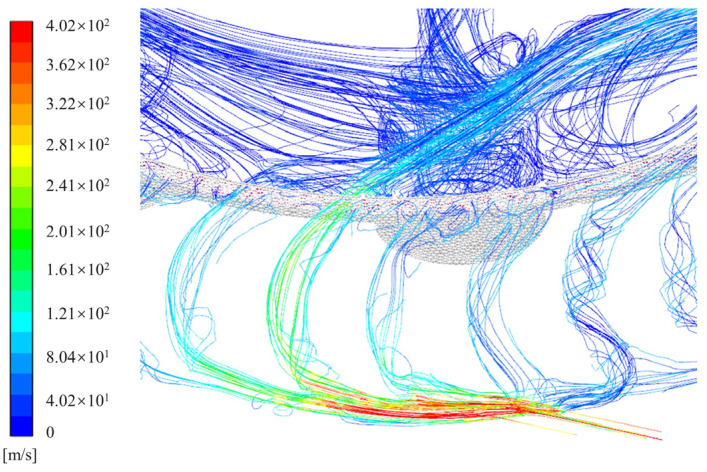
Velocity streamlines within the turbine flow passage.

**Figure 8:**
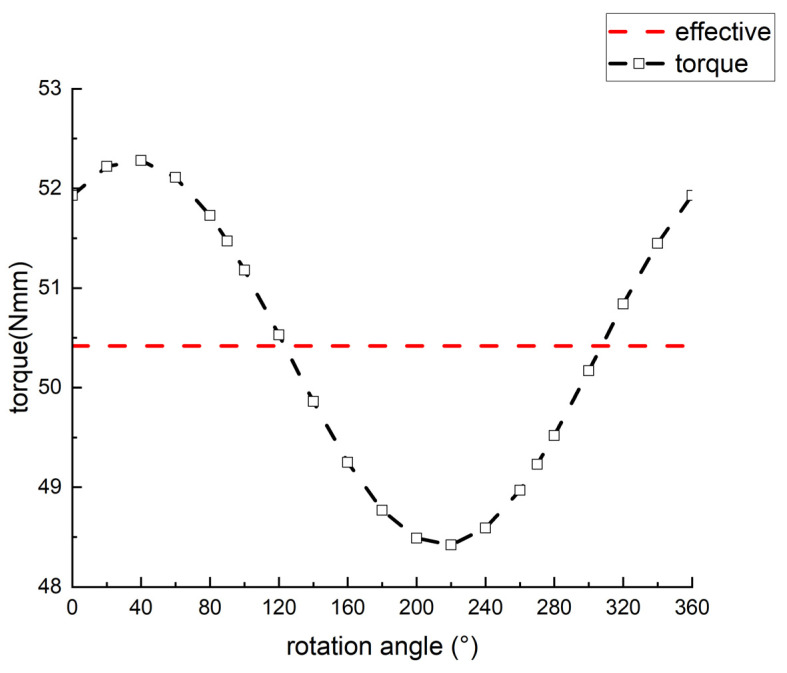
Torque generated by the turbine.

Figure 6 illustrates the pressure distribution within the turbine flow channel. The diagram indicates that the dynamic pressure of the gas within the nozzle is relatively high. However, it experiences a sharp decline in the turbine region. This phenomenon is attributed to the elevated back pressure, or static pressure, in the turbine tooth area, as well as the recirculation caused by the impact of airflow on the turbine teeth. Consequently, it is crucial to design the inner circular arc radius of the turbine teeth and the nozzle angle effectively, as well as to ensure timely discharge of the airflow to minimize the impact of back pressure. This highlights the significant influence of the inner circular arc radius and nozzle angle on the turbine’s rotational speed. Figure 7 shows the streamline diagram of the turbine’s fluid region, indicating that the air reaches its maximum velocity upon exiting the nozzle. Afterward, the airflow impacts the turbine blades and disperses outward. A portion of the airflow flows in the forward direction around the turbine and exits through the nearest exhaust outlet, with minimal impact on turbine vibration. However, another portion of the airflow moves in the reverse direction, forming vortices. This reverse airflow inevitably reduces the turbine’s speed or torque. Therefore, it is essential to appropriately configure the inner circular arc radius of the turbine teeth and the nozzle angle to mitigate the effects of reverse airflow. The torque distribution within the turbine’s fluid region is shown in Figure 8. The results indicate that the average torque generated by the turbine rotor is 50.4 N•mm, meeting the design requirements.

### 3.3. Static Characteristics Analysis of the Bearings

**Figure 9:**
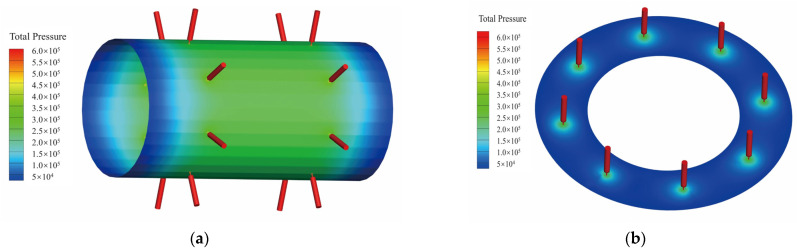
Pressure contour plot of radial and thrust bearings: (**a**) pressure contour map of the radial bearing; (**b**) pressure contour map of the thrust bearing.

**Figure 10:**
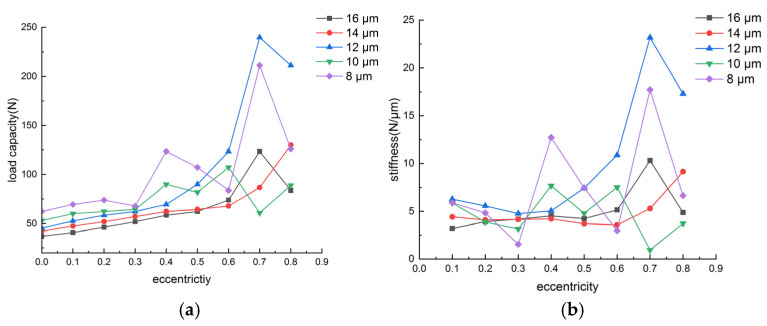
Load performance of the thrust bearings with different eccentricity ratios and film thicknesses: (**a**) load capacity versus eccentricity ratios in different film thicknesses; (**b**) stiffness versus eccentricity ratios in different film thicknesses.

**Figure 11:**
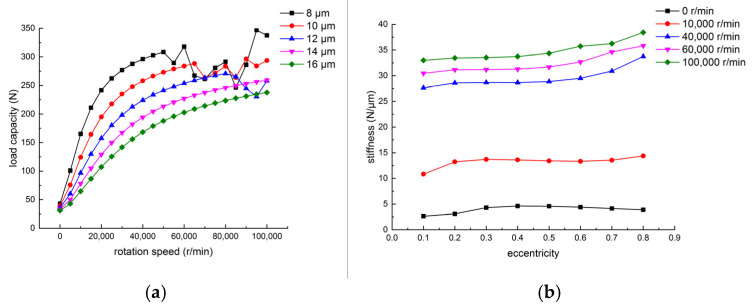
Load performance of the radial bearings with different eccentricity ratios, rotational speeds, and film thicknesses: (**a**) load capacity versus film thickness at different speeds; (**b**) stiffness versus rotational speeds in different eccentricity ratios.

The air-lubricated aerostatic bearings were analyzed through simulations using ANSYS Fluent, as shown in Figure 9. Figure 10 illustrates that, under a supply pressure of 0.6 MPa, the load capacity and stiffness of the thrust bearing decrease with the increasing gas film thickness and increase with the rising eccentricity. Figure 11 shows that, at a supply pressure of 0.6 MPa, the load capacity of the radial bearing increases as the gas film thickness decreases and rotational speed increases, while the stiffness of the gas film in the radial bearing rises with both the eccentricity and rotational speed. The simulation results indicate that, when the supply pressure is 0.6 MPa and the eccentricity is 0.5, the load capacity of the thrust bearing is 89 N, and the load capacity of the radial bearing is 220 N, meeting the load bearing requirements of the air-floating spindle.

## 4. Optimization of the Ultrasonic Rotor Structure and Amplitude Testing

To prevent instability in the ultrasonic rotor at high or ultra-high rotational speeds, which could lead to bearing wear or even rotor seizure, this study employed CFD finite element analysis to examine the structural characteristics of the amplitude transformer and the rotational dynamics of the ultrasonic rotor. This ensures that the spindle rotor can achieve stable high-speed rotation while simultaneously producing small ultrasonic frequency vibrations along the axial direction.

### 4.1. Modal Analysis of the Ultrasonic Amplitude Transformer

The ultrasonic amplitude transformer is a critical component of the ultrasonic spindle responsible for generating vibrations. The design features a composite stepped amplitude transformer with a tool head and a conical transition section, made from 38CrMoAl. The diameter ratio between the input and output ends is 1.87, with the larger end measuring 15 mm in diameter. The acoustic properties of 38CrMoAl are characterized by a density of 7850 kg/m^3^ and a sound velocity of 5470 m/s. The material properties include a Poisson’s ratio of 0.28 and a Young’s modulus of 200 GPa. The amplitude transformer is designed with an amplification factor of 2.92, ensuring efficient vibration transmission.

**Figure 12:**
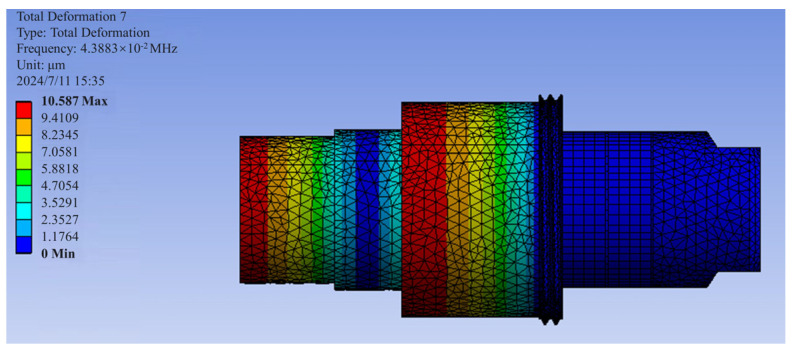
Modal analysis of the ultrasonic horn.

The ultrasonic amplitude transformer has a thread for axial fixation by screwing into the spindle, and radial fixation is achieved through a press fit. A tapered hole is also included to enable tool clamping with a collet. To determine the natural frequencies and vibration modes of the ultrasonic amplitude transformer, a modal analysis was conducted using ANSYS Workbench, with a frequency search range set from 11 kHz to 70 kHz. As shown in Figure 12, the results indicate that the seventh vibration mode is the most suitable for ultrasonic-assisted machining, with a modal frequency of 43.88 kHz. In this mode, the axial end of the transformer exhibits the largest amplitude in the axial direction, while no significant vibration is observed in the radial direction.

**Figure 13:**
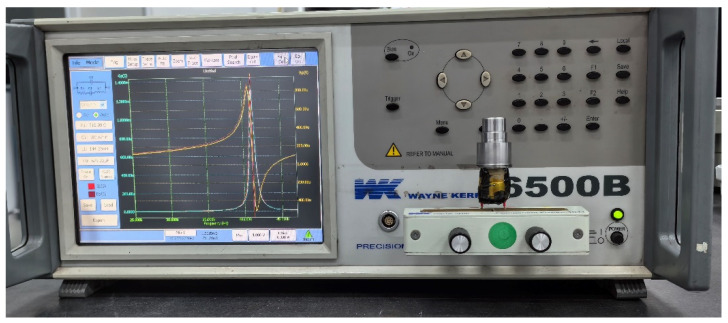
Impedance testing setup for the ultrasonic transducer.

**Figure 14:**
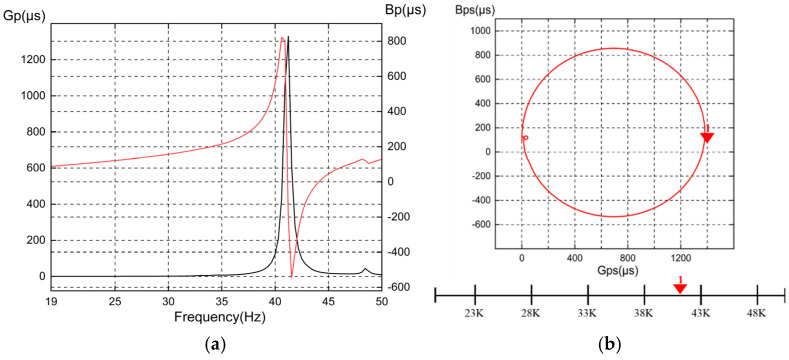
Impedance test results of the ultrasonic transducer: (**a**) impedance analysis results; (**b**) admittance coordinate diagram.

After undergoing appropriate heat treatment, the machined amplitude transformer was assembled with the transducer to form the ultrasonic vibrator. An impedance analyzer (Wayne Kerr 6500B) was used to perform an impedance characteristic analysis, as illustrated in Figure 13. The measurement results are shown in Figure 14. Testing revealed that the resonant frequency of the transducer is 41.23 kHz. The data indicate that the resonant frequencies of the ultrasonic amplitude transformer and the transducer are closely aligned, achieving maximum excitation efficiency, with a frequency deviation of 6.0%.

### 4.2. Dynamic Analysis of the Ultrasonic Spindle Rotor

**Figure 15:**
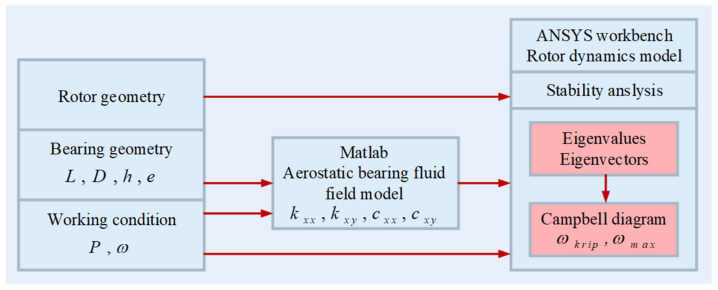
Schematic of the simulation workflow.

The ultrasonic vibrator was fixed to the shaft. By taking into account the gyroscopic effects of the shaft core during rotation, a rotor dynamic model for the hydrodynamic and hydrostatic air-bearing system was established. Using the dynamic stiffness and damping parameters calculated via the finite element method, the natural frequency and critical speed of the ultrasonic rotor were computed. The process flowchart is shown in Figure 15. In the stationary coordinate system, the rotor dynamic equation for the hydrodynamic and hydrostatic air-bearing spindle is expressed as follows [34]:

(10) $$\left[M\right]\left\{\ddot{U}\right\}+(\left[G\right]+\left[C\right])\left\{\dot{U}\right\}+(\left[B\right]+\left[K\right])\left\{U\right\}=\left\{F\right\}$$

where $\left[M\right]$ represents the mass matrix, $\left[G\right]$ is the gyroscopic matrix, $\left[C\right]$ denotes the damping matrix, $\left[B\right]$ is the rotational matrix, $\left[K\right]$ is the stiffness matrix, and $\left\{F\right\}$ represents the external force vector.

**Figure 16:**
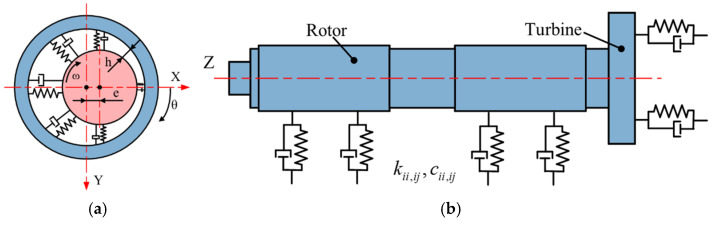
Model of the dynamic air bearing and rotor of the ultrasonic-assisted air bearing spindle: (**a**) model of aerostatic radial bearing; (**b**) equivalent unit of the film rigidity of hybrid air bearing.

The equation is solved as an eigenvalue problem, where the eigenvalues represent the natural frequencies of the rotor system, and the eigenvectors correspond to the modal shapes and the resulting shaft core trajectories. Figure 16 shows the finite element simulation model of the hydrostatic air-bearing spindle system. In this model, the hydrostatic radial bearings are represented by COMBI214 elastic-damping elements, while the hydrostatic thrust bearings are equivalently modeled using COMBIN14 elastic-damping elements [35].

**Figure 17:**
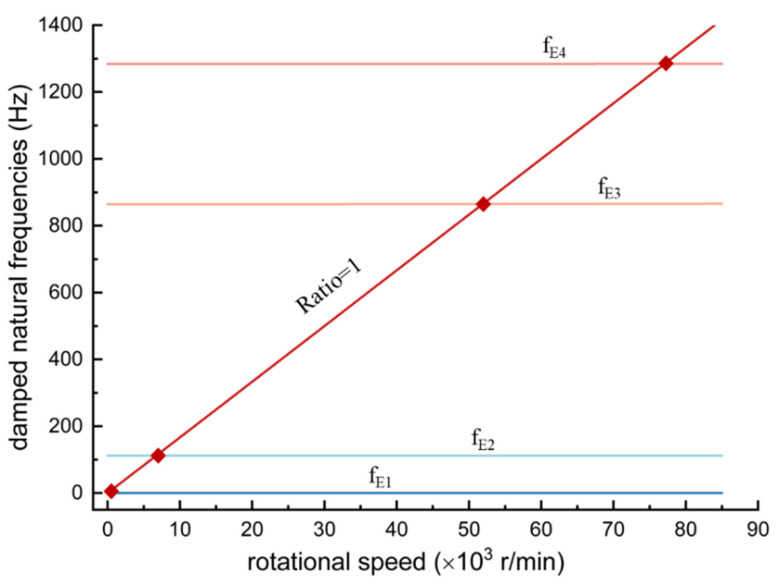
Campbell diagram for the rotor of the ultrasonic-assisted air bearing spindle.

The Campbell diagram in Figure 17 illustrates the rotational frequencies of the spindle rotor within the eigenfrequency domain. When the excitation frequency reaches the rotor’s resonance frequency, the spindle experiences vibrations, leading to contact between the air bearings and the spindle, potentially causing critical damage. The diagram indicates that the critical rotational speeds are 0 r/min, 6733 r/min, 51,883 r/min, and 77,051 r/min. In practical applications, these speeds should be avoided during operation.

### 4.3. Prototype Fabrication and Evaluation

**Figure 18:**
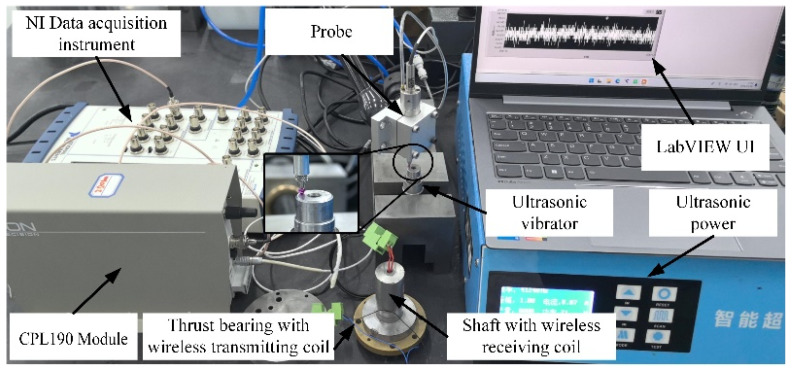
Ultrasonic transducer testing setup.

A prototype wireless inductive power supply device was developed, employing Lion’s CPL190 contact capacitive displacement sensor to evaluate the vibration characteristics of the ultrasonic transducer. The testing setup is presented in Figure 18. The findings demonstrate that the wireless transmission provides stable power, enabling the ultrasonic transducer to achieve high-frequency amplitudes of 0~0.1 μm at 41.23 kHz, meeting the design requirements.

**Figure 19:**
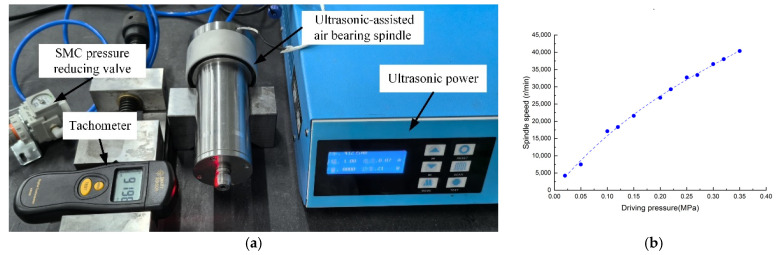
Rotational speed testing setup and results: (**a**) rotational speed testing setup; (**b**) variations of the spindle speed with the driving pressure.

The ultrasonic-assisted air bearing spindle developed in this study has structural dimensions of φ60 mm × 194 mm. Its rotational speed was measured using a tachometer, with the test setup and results displayed in Figure 19. As shown, the spindle speed increases with the rising air supply pressure, reaching 40,000 rpm at a drive pressure of 0.35 MPa, thus meeting the design speed requirements.

## 5. Conclusions

This study presents a comprehensive analysis of an ultrasonic-assisted aerostatic spindle developed for the precision grinding of superhard optical molds. The spindle’s design framework is outlined, with specific attention paid to the optimization of turbine geometry aimed at mitigating vortex effects, yielding a rotor torque of 50.4 N•mm. Computational fluid dynamics (CFD) simulations were employed to investigate the performance of the spindle’s radial and axial gas-static bearings. Under an air supply pressure of 0.6 MPa and an eccentricity ratio of 0.5, the simulations demonstrate a thrust bearing load capacity of 89 N and a radial bearing capacity of 220 N, indicating adequate load-bearing performance.

Subsequently, finite element analysis was employed to optimize the structure of the ultrasonic amplitude transformer, followed by an analysis of the dynamic characteristics of the shaft core. Finally, the impedance characteristics of the transducer, vibration properties of the ultrasonic transducer, and spindle speed were tested. The analysis results indicate that the ultrasonic spindle achieves a rotational speed of up to 40,000 rpm and generates high-frequency vibrations at 41.23 kHz, meeting the design specifications. Additionally, critical speeds, such as 6733 r/min, 51883 r/min, and 77,051 r/min, should be avoided to prevent spindle vibrations during operation.

For further research, subsequent machining tests will be conducted to assess the spindle’s effectiveness in ultra-hard optical mold processing. Furthermore, the spindle structure will undergo additional optimization to maximize the rotational speed and enhance the ultrasonic performance.

## Figures and Tables

**Table 1:** Material properties of PZT-8 ceramic materials.

Electromechanical Coupling Coefficient $K_{t}$	Piezoelectric Constant $d_{33}(10^{-12}$ C/N)	Mechanical Quality Factor $Q_{M}$	Dielectric Parameter $\sum_{33}^{t}/\sum_{o}$	Dielectric Loss tgδ%	Curie Point Tc (°C)	Density ρ $\;10^{3}(kg/$ $m^{3})$	Elastic Modulus $10^{10}\left(Nm^{-2}\right)$	Poisson Ratio σ
0.45	232	1200	970	0.29	306	7.5	7.65	0.32

## Data Availability

Data are contained within the article.
